# Pulsed Electromagnetic Field Therapy and Direct Current Electric Field Modulation Promote the Migration of Fibroblast-like Synoviocytes to Accelerate Cartilage Repair In Vitro

**DOI:** 10.3390/app122312406

**Published:** 2022-12-04

**Authors:** Neeraj Sakhrani, Robert M. Stefani, Stefania Setti, Ruggero Cadossi, Gerard A. Ateshian, Clark T. Hung

**Affiliations:** 1 Department of Biomedical Engineering, Columbia University, New York, NY 10027, USA; 2 Igea Clinical Biophysics, 41012 Carpi, Italy; 3 Department of Mechanical Engineering, Columbia University, New York, NY 10027, USA; 4 Department of Orthopedic Surgery, Columbia University, New York, NY 10032, USA

**Keywords:** pulsed electromagnetic fields, galvanotaxis, DC electric fields, synovium, migration, in vitro models

## Abstract

Articular cartilage injuries are a common source of joint pain and dysfunction. As articular cartilage is avascular, it exhibits a poor intrinsic healing capacity for self-repair. Clinically, osteochondral grafts are used to surgically restore the articular surface following injury. A significant challenge remains with the repair properties at the graft-host tissue interface as proper integration is critical toward restoring normal load distribution across the joint. A key to addressing poor tissue integration may involve optimizing mobilization of fibroblast-like synoviocytes (FLS) that exhibit chondrogenic potential and are derived from the adjacent synovium, the specialized connective tissue membrane that envelops the diarthrodial joint. Synovium-derived cells have been directly implicated in the native repair response of articular cartilage. Electrotherapeutics hold potential as low-cost, low-risk, non-invasive adjunctive therapies for promoting cartilage healing via cell-mediated repair. Pulsed electromagnetic fields (PEMFs) and applied direct current (DC) electric fields (EFs) via galvanotaxis are two potential therapeutic strategies to promote cartilage repair by stimulating the migration of FLS within a wound or defect site. PEMF chambers were calibrated to recapitulate clinical standards (1.5 ± 0.2 mT, 75 Hz, 1.3 ms duration). PEMF stimulation promoted bovine FLS migration using a 2D in vitro scratch assay to assess the rate of wound closure following cruciform injury. Galvanotaxis DC EF stimulation assisted FLS migration within a collagen hydrogel matrix in order to promote cartilage repair. A novel tissue-scale bioreactor capable of applying DC EFs in sterile culture conditions to 3D constructs was designed in order to track the increased recruitment of synovial repair cells via galvanotaxis from intact bovine synovium explants to the site of a cartilage wound injury. PEMF stimulation further modulated FLS migration into the bovine cartilage defect region. Biochemical composition, histological analysis, and gene expression revealed elevated GAG and collagen levels following PEMF treatment, indicative of its pro-anabolic effect. Together, PEMF and galvanotaxis DC EF modulation are electrotherapeutic strategies with complementary repair properties. Both procedures may enable direct migration or selective homing of target cells to defect sites, thus augmenting natural repair processes for improving cartilage repair and healing.

## 1. Introduction

Articular cartilage is a highly specialized connective tissue that overlies the surface of bones in diarthrodial joints [1,2]. It provides a smooth, lubricated surface to minimize friction during articulation and distributes mechanical forces across the joint capsule to protect the underlying subchondral bone [1,3]. While articular cartilage functions to absorb compressive loads, applied mechanical stress contributes to gradual wear, tears, and injuries to the joint [2,4]. However, articular cartilage exhibits a poor intrinsic healing capacity for self-repair, as it is avascular, aneural and alymphatic [1,2,5]. Without targeted therapies and repair treatment strategies, articular cartilage damage can contribute to gradual tissue deterioration, debilitating pain, joint inflammation, and ultimately complete degradation in the pathogenesis of osteoarthritis (OA) [2,6].

The poor regenerative capacity of articular cartilage necessitates effective treatment methods to initiate joint healing and repair. Transplantation of cartilage osteochondral grafts, autologous or allogeneic depending on lesion size, is a common operative treatment to repair cartilage defect areas [7,8]. However, a significant challenge remains with the integrative repair properties at the graft-host tissue interface, as proper surgical implantation and integration to the subchondral bone are critical toward restoring normal load distribution across the joint [9,10]. Osteochondral grafts may also undergo central necrosis and appearance of subchondral cyst-like resorption areas, which may contribute to graft mechanical instability, poor cartilage nutrition, and ultimately graft failure [10]. Several cell-based treatments, including autologous chondrocyte implantation (ACI) and mesenchymal stem cell (MSC) delivery therapies, have emerged as surgical options for cartilage repair [11]. Both procedures seed cells that directly participate in defect healing and regulate cartilage metabolism by producing extracellular matrix (ECM) molecules that are critical for native function, including collagens, proteoglycans, and glycosaminoglycans (GAGs) [12,13]. However, ACI and MSC delivery to defect lesions in the joint possess clinical limitations, including risk of further injury to healthy cartilage during autologous harvest, the need for two-step surgical techniques, cellular immune rejection following implantation, and graft hypertrophy [14,15]. Instead of surgical interventions, natural repair processes via direct migration or selective homing of native target cells to defect sites in the cartilage surface hold potential as alterative treatment strategies [13,16]. Articular cartilage is composed of chondrocytes, embedded within a dense extracellular matrix of collagens and proteoglycans [1,5]. However, native chondrocytes have limited metabolic activity, proliferation, and biosynthesis, contributing to poor healing response following joint injury [1,17]. Therefore, in order to address poor tissue integration and limited chondrocyte migration, fibroblast-like synoviocytes (FLS) that exhibit chondrogenic potential may serve as a cell-based strategy to promote in vitro cartilage repair. FLS are derived from the adjacent synovium, the specialized connective tissue membrane that envelops the diarthrodial joint [18–20]. Synovium-derived cells have been directly implicated in both the native repair response as well as degradation of articular cartilage [21], thus providing a promising target for the development of novel strategies aimed toward preventing structural changes to the joint and treating clinical symptoms, especially in the development of early OA [22]. The proposed studies have been inspired by in vivo lineage tracing experiments in mice where the preponderance of evidence (labeled cells from three independent mouse strains including postnatal tamoxifen induction, proliferative capacity of this cell lineage, no migration of adjacent articular chondrocytes) suggests that cells from the synovium are the source for healing [23,24]. These studies suggest repair cells migrating into the full thickness cartilage defects are primarily derived from the synovium rather than bone marrow or adjacent cartilage [23,24]. Therefore, strategies aimed at targeting synovial cells could offer novel and effective therapies to boost repair and regeneration of joint tissues [23].

The poor intrinsic healing ability of articular cartilage is predominantly attributed to scarce migration of native cells that have the potential to repair the defect site [25]. Electrotherapeutics possess potential as low-cost, low-risk, and non-invasive adjunctive therapies for improving the rate of articular cartilage repair [26,27]. Electrical stimulation of native cells within larger tissue and organ systems is promising as endogenous electric field (EF) gradients have been shown to guide cellular behavior during wound healing [28,29]. Pulsed electromagnetic fields (PEMFs) and applied direct current (DC) EFs via galvanotaxis are two potential electrotherapeutic strategies that have yet to be fully explored in the context of promoting cartilage repair by stimulating the migration of native FLS within the wound or defect site.

PEMF treatment has been clinically used to stimulate bone repair and alleviate joint pain [30]. Studies have shown that PEMF stimulation is an adjuvant, anti-inflammatory therapy and pain management tool that contributes to improvements in joint function as well as pain relief following arthroscopy, including microfracture, chondroabrasion, collagen scaffold seeded with bone marrow–derived cells (BMDCs), matrix-assisted chondrocyte implantation (MACI), osteochondral allograft (OCA) transplantation, and knee arthoplasty [31–34]. However, the use of PEMF treatment for articular cartilage repair has not been thoroughly investigated yet, with limited mechanistic studies characterizing the application of this electrotherapeutic technique in initiating an intrinsic healing response [35,36]. Previous work in our laboratory demonstrated the positive impact of PEMF on articular cartilage defect repair in vivo with engineered cartilage constructs [37]. The current study develops an in vitro model, where experimental boundary conditions are well defined, to gain a better understanding of how PEMFs can be used to directly augment natural cartilage repair strategies.

DC EFs is another electrotherapeutic strategy that may stimulate the intrinsic repair process of synovium and articular cartilage. EFs of strengths from 1 to 10 V/cm have been shown to induce directed movement (galvanotaxis) and shape change (galvanotropism) in several musculoskeletal cell types, including chondrocytes, osteoblasts, and meniscal fibrochondrocytes [38–41]. Endogenously generated gradients within this range of EF strengths have also been shown to guide cell migration at the cut surface of wounds [29,42]. Clinically, the placement of electrodes for galvanotaxis may involve slightly invasive interventions compared to PEMF systems. Nonetheless, similar to PEMFs, DC EF galvanotaxis may also serve as a useful tool for promoting articular cartilage repair via directed FLS migration toward the defect region.

FLS migration in 2D is yet to be fully explored in the context of promoting intrinsic cartilage repair under electrotherapeutic stimulation. Study 1 characterized the migration of bovine FLS using an in vitro scratch assay under PEMF exposure to assess the rate of wound closure following cruciform injury. To further elucidate the electrotherapeutic potential for synovial cell-mediated repair, study 2 investigated the effect of EF stimulation via galvanotaxis to direct bovine FLS migration within a collagen hydrogel matrix.

The benefits of electrotherapeutic strategies on cartilage and synovium explants within an in vitro defect-repair model have also not been investigated. Therefore, studies were translated into the three-dimensional tissue environment to track FLS migration from healthy bovine synovium to injured cartilage explants. By developing and validating a novel tissue-scale bioreactor capable of applying DC EFs in sterile culture conditions to 3D constructs, study 3 investigated the recruitment of synovial repair cells via galvanotaxis from intact synovium explants to the site of a cartilage wound injury. Similarly, study 4 characterized the effect of PEMF stimulation on modulating the migration of endogenous and/or exogenous FLS from the bovine synovial membrane into the adjacent cartilage defect.

Limited studies characterizing the functional outcomes and intrinsic biochemical properties of both synovium and cartilage explants under electrotherapeutic stimulation have been performed [37]. Therefore, study 5 assessed the biochemical composition of bovine synovium explants and injured cartilage constructs via DNA, GAG, and collagen content following PEMF treatment. Histological characterization of both explants was evaluated in order to confirm differences observed from the functional biochemical assays. Gene expression of cartilage and synovium ECM constituents, specifically collagens, were assessed in order to characterize the recruitment of chondrocytes and FLS into the bovine cartilage defect region following PEMF stimulation.

PEMF and galvanotaxis DC EF modulation are potential complementary strategies that may enhance FLS migration, providing alternative nonoperative methods to accelerate cartilage repair following injury [43,44]. The purpose of the current study is to investigate the potential of both techniques in initiating an intrinsic healing response by stimulating FLS movement toward injured articular cartilage lesions, which may lead to peripheral cellular integration along the defect site and ultimately repair the joint surface with similar biochemical properties and mechanical characteristics as healthy hyaline cartilage [45].

## 2. Materials and Methods

### 2.1 PEMF System

**Figure 1.**
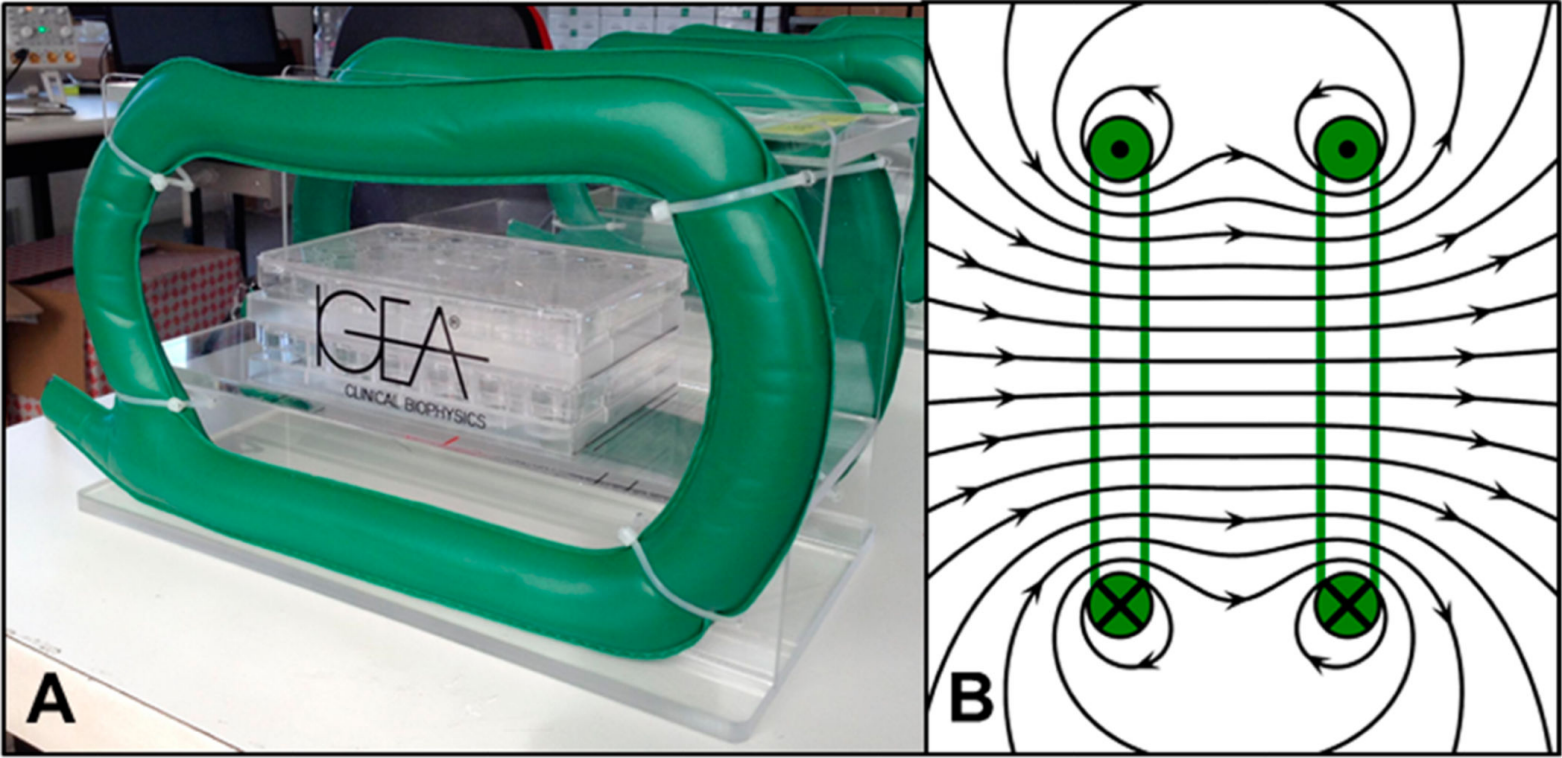
In vitro PEMF chamber consisting of (**A**) two electromagnetic Helmholtz coils (green) that create a (**B**) uniform magnetic field in the culture region.

Custom PEMF generators were assembled by IGEA Clinical Biophysics (Carpi, Italy). The generators were constructed using two Helmholtz coils made of copper wire on opposite sides of a plexiglass chamber (Figure 1A). A signal generator was used to create a uniform magnetic field within the culture area (Figure 1B). The PEMF system was calibrated to clinical standards with a 1.5 ± 0.2 mT peak intensity of magnetic field, 1.3 ms pulse duration, and 75 Hz frequency [37]. The magnetic field intensity within the chamber was determined using a gaussmeter (Walker Scientific, Auburn Hills, MI, USA) with a sensitivity reading of 0.2% and the induced electric field was measured using a coil probe (50 turns). A digital oscilloscope (Le Croy, Chestnut Ridge, NY, USA) was used to characterize the temporal pattern of the signal to ensure that the shape and impulse length of the generated electric field remain constant [37].

### 2.2. Galvanotaxis Chamber for 2D Culture

**Figure 2.**
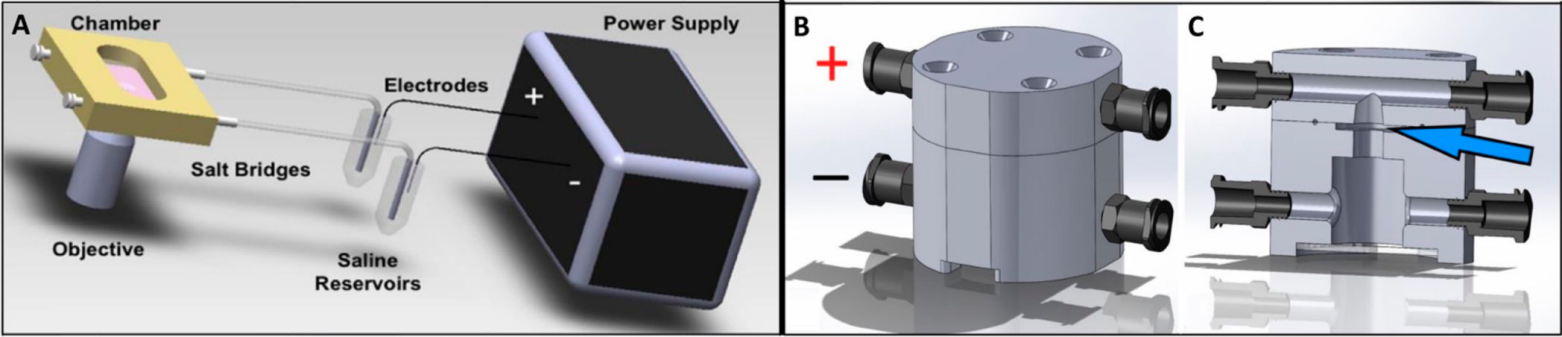
(**A**) Schematic of classic 2D galvanotaxis system. Cells are plated on a glass slide within a custom chamber and exposed to EF gradient for three hours. (**B,C**) Design of novel 3D galvanotaxis chamber with denoted electrode positions (+ and −). The system allows EF gradients to be applied to 3D tissues and constructs (blue arrow denotes sample placement within the chamber).

The galvanotaxis system (Figure 2A) was constructed following a flow chamber assembly [25]. Silver–silver chloride electrodes were fabricated from silver wire and soaked in a hypochlorite solution (Clorox Bleach) for 1 h. Electrodes were connected to ports on both sides of the galvanotaxis channel using a pair of 2% agarose–PBS bridges. These bridges prevented cell contamination from electrolysis products generated during EF stimulation using a power supply (Keithley Instruments, Cleveland, OH, USA), which delivered a current of 3.3 mA (6 V/cm EF strength) [25]. The conductive media that flowed through the galvanotaxis chamber system was Dulbecco’s Modified Eagle’s Medium (DMEM; Cat. No. 12100046; Thermo Fisher Scientific, Waltham, MA, USA) supplemented with 5% fetal bovine serum (FBS; Cat. No. S11550; R&D Systems, Minneapolis, MN, USA), which had adequate protein concentration for cellular adherence as well as ion concentrations for current flow. Galvanotaxis DC EF stimulation was applied for 3 h at room temperature. The chamber also consisted of a glass panel, allowing for microscopic analysis of cellular migration.

### 2.3. Galvanotaxis Chamber for 3D Culture

Using the mechanism of the 2D galvanotaxis system (Figure 2A), a novel 3D galvanotaxis chamber (Figure 2B,C) was designed using CAD software (Solidworks, Waltham, MA, USA). The upper and lower compartments of the chamber was separated by an O-ring and secured together with screws, creating a sealed cylindrical channel with a 5 mm diameter and a 1 mm height. This internal chamber accommodated the culture of cylindrical specimens while preventing current leakage. The dimensions and well-defined chamber geometry of the 3D galvanotaxis system allowed for the applied electric field strength (E) and current density (J) to be calculated using Ohm’s Law. The calculated resistance of the chamber was 35.8 kΩ, which was consistent with clinical applications. The anode (+) and cathode (−) were positioned above and below the cylindrical culture region. The total chamber volume was approximately 2.5 mL, allowing sufficient media supplementation for constructs and ion concentration to conduct current.

The galvanotaxis chamber was 3D printed using an Ultimaker S5 (Cura v4.2.1; Utrecht, The Netherlands) with Taulman Nylon 680 filament. Nylon filament was selected due to FDA approval and compatibility with autoclaving and ethanol sterilization. The chamber assembly was tapped and drilled prior to sealing the chamber with a clear acrylic cap for the bottom inlet of the bioreactor system. The external power supply, electrodes, and salt bridges were connected to the 3D galvanotaxis chamber and prepared similar to the 2D chamber system.

### 2.4. Bovine Synovium and Cartilage Explant Harvest

**Figure 3.**
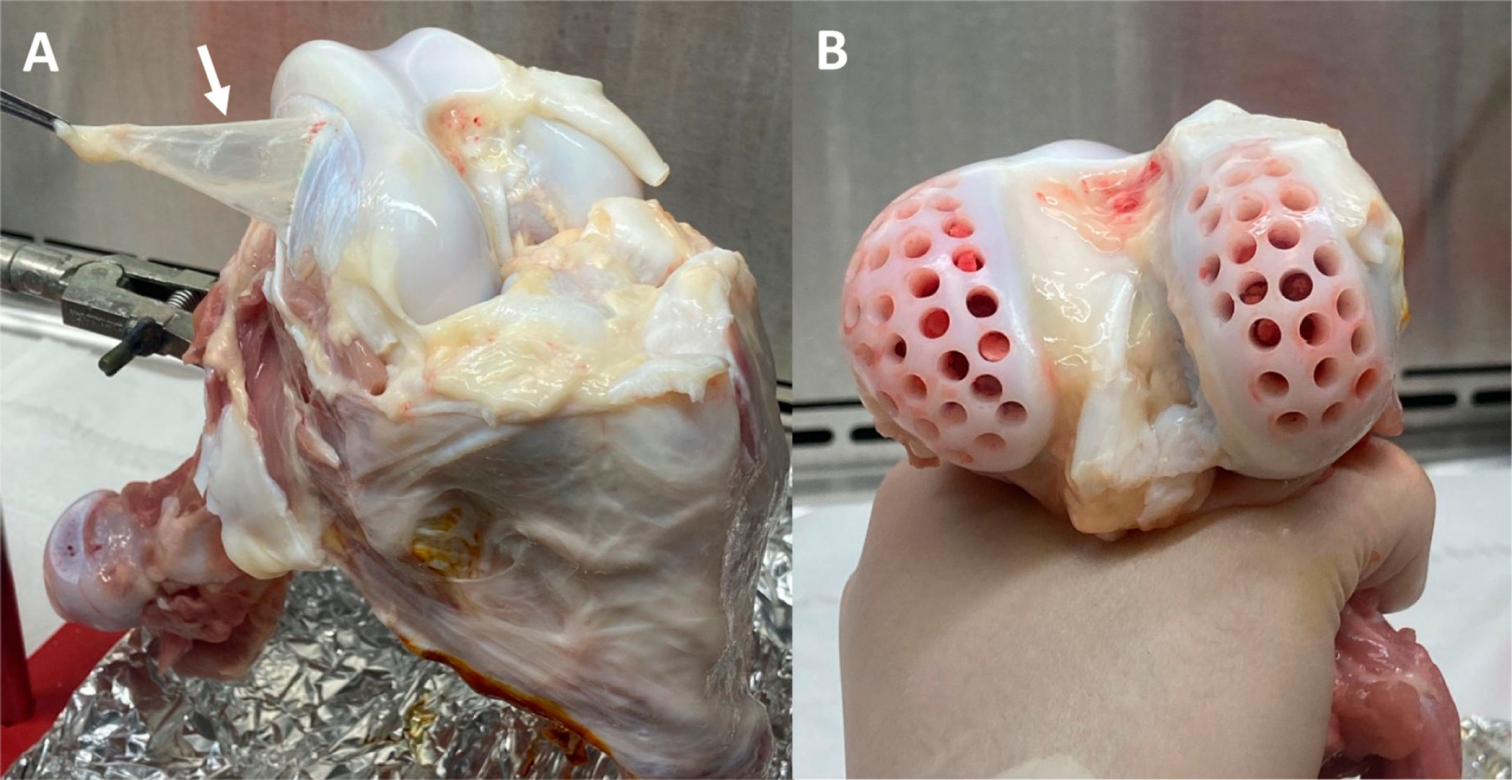
Juvenile bovine knee explant harvest. (**A**) Synovium (arrow) was harvested from the medial and lateral femoral condyles of the joint. (**B**) Osteochondral grafts were collected using a 4 mm biopsy punch from the femoral condyle.

Fresh synovial explants (Figure 3A) and cartilage plugs (Figure 3B) were harvested from discarded (IACUC-exempt) bovine calf knee joints (2–4 weeks old) [46]. The thin synovial sheath was extracted from the region adjacent to the medial and lateral femoral condyles. Synovium explants from 3 bovine knee joints were combined and cut into consistent ~1 cm × 1 cm pieces. Osteochondral dowel grafts were harvested using a 4 mm diameter dermal trephine biopsy from the femoral condyle of the joint. The acquired osteochondral plugs were cut 2 mm through the depth of the construct, from the articular cartilage surface to the deep cartilaginous zone, removing the subchondral bone layer. Prior to studies, synovium explants and cartilage plugs were cultured for five days in serum-free media consisting of DMEM supplemented with 50 μg/mL L-proline (Cat. No. P5607; Sigma-Aldrich, St. Louis, MO, USA), 100 μg/mL sodium pyruvate (Cat. No. S8636; Sigma-Aldrich, St. Louis, MO, USA), 1% ITSTM+ Premix (contains insulin, transferrin, selenous acid, BSA, and linoleic acid; Cat. No. 354352; Corning, Corning, NY, USA), 1% antibiotic–antimycotic (AA; Cat. No. 15240062; Thermo Fisher Scientific, Waltham, MA, USA), and 50 μg/mL ascorbic acid-2-phosphate (Cat. No. A8960; Sigma-Aldrich, St. Louis, MO, USA).

### 2.5. Bovine FLS and Chondrocyte Isolation

For 2D studies, bovine synovium and articular cartilage explants were separately digested using collagenase type II (Cat. No. LS004177; Worthington Biochemical Corporation, Lakewood, NJ, USA) with gentle stirring at 37 °C for 4 and 11 h, respectively. Digested synovial fibroblasts and articular chondrocytes were filtered through a sterile 70 μm porous nylon mesh to remove any residual explant specimens. Viable FLS were counted and expanded using α-Minimum Essential Medium (αMEM; Cat. No. 12000022; Thermo Fisher Scientific, Waltham, MA, USA) containing 10% FBS, 1% AA, and 5 ng/mL fibroblast growth factor-2 (FGF-2; Cat. No. PHG0264; Thermo Fisher Scientific, Waltham, MA, USA) [21,25,46,47]. Articular chondrocytes were grown to confluency using DMEM supplemented with 10% FBS, and 1% AA, and 50 μg/mL ascorbic acid-2-phosphate. FLS and chondrocytes were both expanded for two passages to obtain a pure population of cells. MSC markers (with no expression of endothelial cells) were previously confirmed in cells isolated using this procedure [21,25].

### 2.6. Wound Closure Assay with PEMF Stimulation

Wound closure assay was performed to characterize FLS migration via PEMF stimulation following injury. Bovine FLS were seeded in two separate 12-well plates at 0.1 × 10^6^ cells/well and expanded until confluent using αMEM supplemented with 10% FBS, 1% AA, and 0.5 ng/mL FGF-2. FLS were inflicted with a cruciform wound using a P20 pipette tip. Following injury, one plate was cultured within the PEMF chamber, which was active for 8 h per day, while the other plate received 0 h of electrical stimulation (sham) [37]. Resulting images of the wound were assessed at 0, 12, 24, and 48 h (Olympus IX-70 inverted microscope) following the initial cross-shaped scratch. Images were processed in ImageJ (convert to 8-bit, invert black and white) and percent closure area was quantified for both PEMF treated and sham FLS groups (*n* = 6 wells per treatment group).

### 2.7. FLS Migration in Collagen Hydrogel with DC EF Stimulation

Bovine FLS were encapsulated in a type I collagen hydrogel (2 mg/mL; Cat. No. A1064401; Thermo Fisher Scientific, Waltham, MA, USA) at a seeding density of 10^5^ cells/mL, yielding a thin collagen gel with the exact geometry of the galvanotaxis channel. The concentrated collagen mixture was osmotically balanced using 10X PBS and neutralized with 1 N NaOH before cell integration. Prior to DC EF exposure, the FLS-seeded collagen hydrogel was allowed to solidify directly in the galvanotaxis chamber for 2 h at 37 °C.

Photomicrographs of galvanotaxis DC EF stimulation were acquired at 10 min intervals throughout the 3 h stimulation period. Images were manually analyzed using custom MATLAB code in order to determine the overall and incremental speeds, migration direction, and directed velocity of the FLS-seeded collagen gel. The centroid of each cell was monitored, where the initial starting position was considered the coordinates of the origin [39]. Overall migration speed was derived from the net displacement (i.e., magnitude of the vector starting at the origin and ending at the final cell position) divided by the 3 h stimulation time. Incremental speed was computed for all photomicrographs following each 10 min acquisition interval. Migration direction was determined by the orientation of the net displacement vector, whereby the cathode (−) and anode (+) were positioned at 90° and 270°, respectively. Directed velocity was defined as the speed component directed toward the cathode [25]. Mean migration angle was determined by calculating the average of all unit vectors over the 10 min interval for each cell. A total of 20 cells were analyzed for each DC EF stimulation trial (*n* = 3).

### 2.8. FLS Migration into a Cartilage Defect

**Figure 4.**
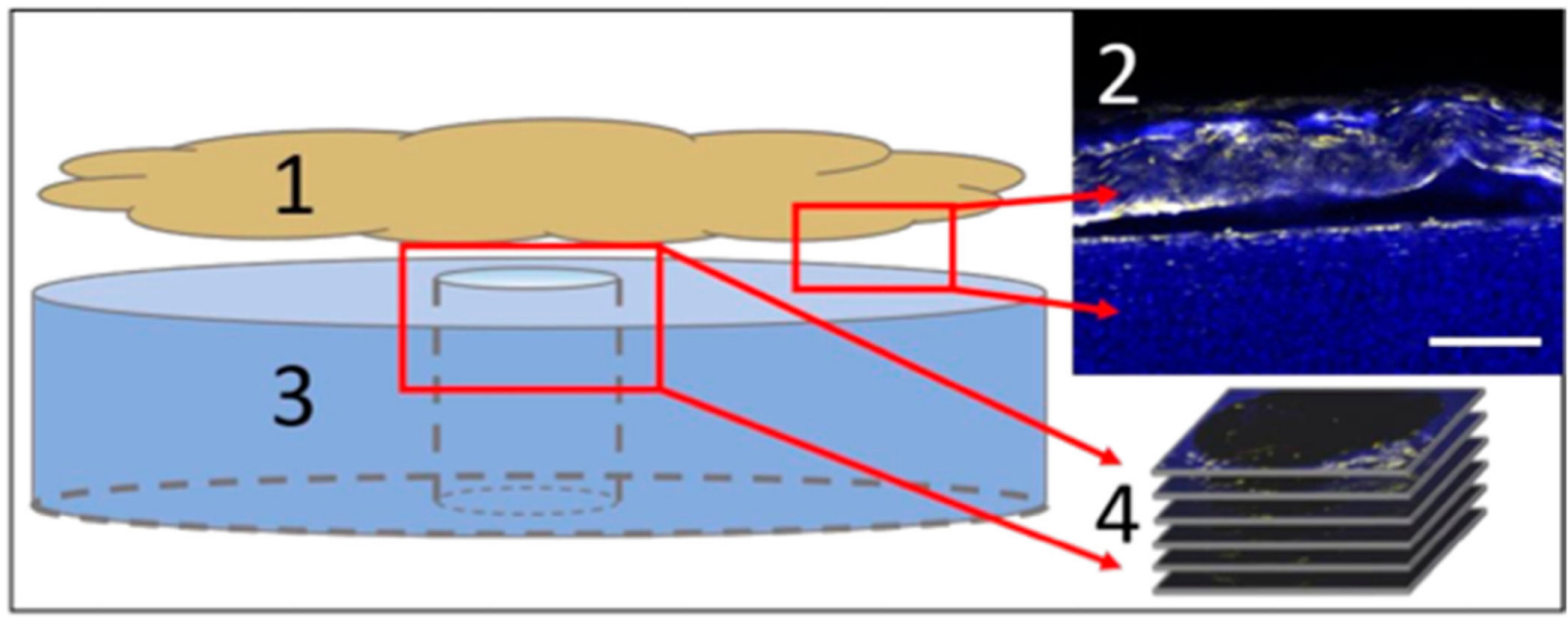
Experimental set-up of cartilage-synovium defect-repair model. DiI-labeled synovium (1, yellow) was placed in direct apposition (2) with DAPI-labeled cartilage (3, blue). Synovium was removed for confocal z-stack imaging (4), leaving the DiI-stained FLS on cartilage specimen. Migration of FLS were monitored through the depth of the cartilage defect annulus. Scale bar = 100 μm.

To form a cartilage defect, 1 mm cores were removed from each 4 mm bovine cartilage plug using a trephine biopsy. The obtained ~1 cm × 1 cm pieces of bovine synovium were stained using a lipophilic membrane dye (Vybrant DiI; Life Technologies, Carlsbad, CA, USA). Synovium samples were oriented in direct apposition to the cartilage explant (Figure 4), covering the 1 mm defect region, prior to PEMF or DC EF stimulation. The cartilage plug with the overlying synovium sheath was cultured in DMEM supplemented with 5% FBS, 1% AA, and 50 μg/mL ascorbic acid-2-phosphate. Explants were separately placed in the PEMF and galvanotaxis chambers (*n* = 6). Control samples were handled similarly, but without the application of electrical stimulation. PEMF chamber was active for 8 h per day and explants were treated for 48 h [37]. The galvanotaxis EF current was applied for 3 h, generating an applied field strength of E = 6 V/cm (EF) [25].

Following PEMF and DC EF stimulation, explants were fixed in 4% paraformaldehyde overnight. The synovium was removed from the cartilage construct, leaving behind migrated synovial cells. Cartilage plugs were subsequently stained with DAPI (Sigma-Aldrich, St. Louis, MO, USA) for co-localization of endogenous cartilage cells with the DiI stained synovial cells in the sub-cored defect region. Confocal microscopy (Zeiss, Oberkochen, Germany) was used to visualize synovial cell migration through the depth of the cartilage construct. Z-stacks with a 40 μm step-size were obtained in order to track the migration distance from the articular cartilage surface across the defect site (Figure 4). A 40 μm step-size was selected to ensure that no FLS were missed or double counted during image processing and cell counting analysis. Only FLS that had adhered to the periphery of the cartilage construct were counted for migratory behavior, which was assessed by the co-localization of DAPI and DiI stains along the cartilage surface. FLS were classified as “transferred” if cells were in contact with the topmost layer of the defect site (visible in first z-stack image). FLS were considered “migrated” if cells were visible at any location ≥40 μm though the cartilage depth. The number of migrated cells was normalized to the total FLS count across the entire cartilage construct for both PEMF and galvanotaxis treated specimens. For explants exposed to DC EF stimulation, directed FLS velocity was computed assuming constant cell migration throughout the 3 h stimulation period. Transferred or non-migrating cells that remained on the cartilage surface were considered to have a velocity of 0 μm/h.

### 2.9. Biochemistry

Following PEMF treatment, bovine synovial and cartilage explants (*n* = 6) were frozen at −20 °C and lyophilized overnight. Lyophilized explants were weighed to obtain dry weight measurements prior to tissue digestion. Samples were solubilized by incubating for 16 h at 56 °C in 0.5 mg/mL Proteinase K (Cat. No. 193504; MP Biomedicals, Irvine, CA, USA) and Proteinase K buffer solution containing 50 mM Tris saline, 1 mM EDTA, 1 mM iodoacetamide (Cat. No. 12227–1000; Acros Organics, Geel, Belgium), and 10 mg/mL pepstatin A (Cat. No. BP2671100; Thermo Fisher Scientific, Waltham, MA, USA) [21,47]. DNA content was analyzed using Picogreen (Cat. No. P11496; Thermo Fisher Scientific, Waltham, MA, USA) quantitation assay. GAG levels were measured using a 1,9-dimethylmethylene blue dye-binding assay (Product No. 341088; Sigma-Aldrich, St. Louis, MO, USA) and collagen content was quantified via orthohydroxyproline (OHP) assay with a 1:7.64 OHP-to-collagen mass ratio [47].

### 2.10. Histological Characterization

PEMF treated bovine synovium and cartilage samples were fixed using 4% paraformaldehyde. Specimens were embedded in paraffin wax and sectioned into 4-μm slices. Deparaffinized sections were stained with hematoxylin and eosin (H&E) to determine FLS and chondrocyte distribution within the 1 mm sub-cored region. Synovium and cartilage explants were also stained with Safranin O to assess GAG content and Picrosirius Red to characterize collagen distribution.

### 2.11. qPCR Preparation

Total RNA was extracted from bovine cartilage explants following PEMF exposure for 7 days, with parallel untreated controls. Cartilage was homogenized using TRIzol reagent (Cat. No. 15596018; Thermo Fisher Scientific, Waltham, MA, USA) and chloroform was added to extract total RNA followed by vigorous agitation [48]. RNA precipitation was performed using Qiagen miRNeasy columns (Cat. No. 74106; QIAGEN, Hilden, Germany). cDNA was synthesized from RNA using the iScript cDNA Synthesis Kit (Product No. 1708891; Bio-Rad, Hercules, CA, USA) and diluted to 1 ng/μL concentration for PCR. Gene specific master mixes were prepared using the designed primers, and iTaq Universal SYBR Green Supermix (Product No. 1725122; Bio-Rad, Hercules, CA, USA). RT-qPCR was run on a QuantStudio^™^ 6 Flex Real-Time PCR System (Applied Biosystems, Waltham, MA, USA) [47]. The following primers (Integrated DNA Technologies) were evaluated: aggrecan (ACAN) and collagen types I-V, X-XI alpha 1 chain (COL1, COL2, COL3, COL4, COL5, COL10, COL11) (Table 1). GAPDH was used as the housekeeping gene, and all samples were normalized to day 0 controls using the 2^−ΔΔCt^ method.

### 2.12. Statistics

Normality and homogeneity of the acquired data were assessed using the Kolmogorov–Smirnov Test and Bartlett’s Test, respectively. Non-normal data was log-transformed to achieve normality for subsequent parametric tests. Data sets were analyzed using one-way ANOVA with Tukey post hoc test (α = 0.05). Statistical analysis was performed in GraphPad Prism 9 (La Jolla, CA, USA). Values presented as mean *±* standard deviation.

## 3. Results

### Study 1. Assess effect of PEMF on FLS wound closure in a 2D injury model.

**Figure 5.**
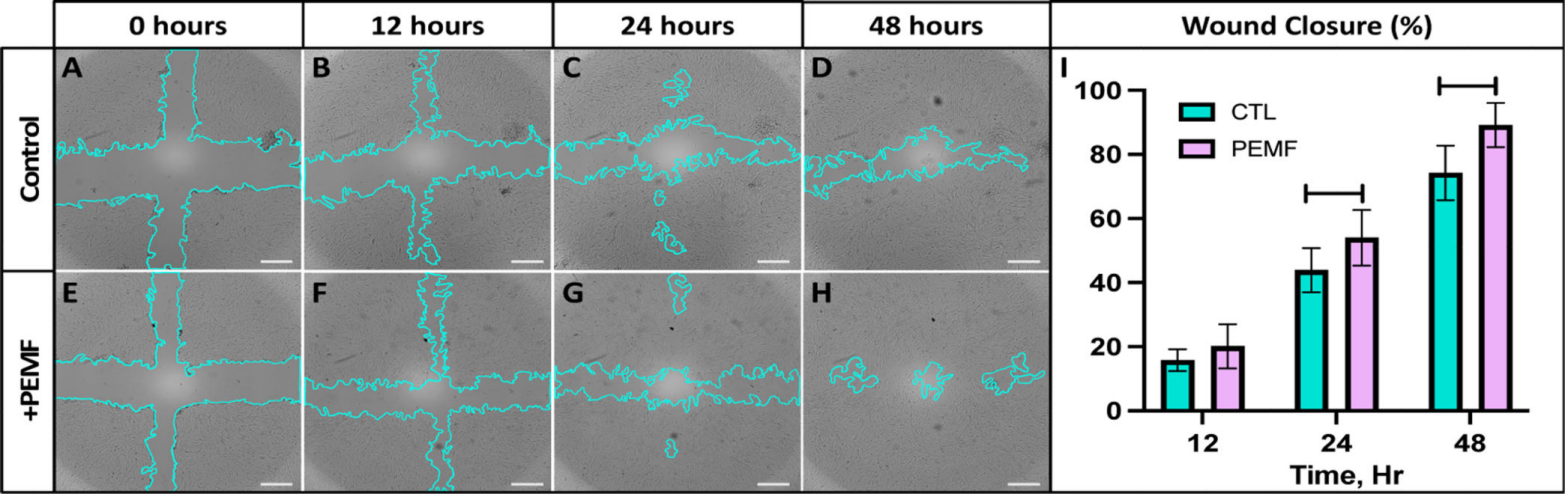
Representative images from in vitro wound healing assay demonstrating FLS migration into the cell-free region (blue) for (**A–D**) control and (**E–H**) PEMF treated samples (Scale bar = 100 μm). (**I**) Quantified percent wound closure of control and PEMF exposed FLS at 12, 24, and 48 h following initial cruciform injury (*n* = 6 per treatment condition).

A scratch assay using a monolayer of bovine FLS was used to determine if PEMF stimulation could promote cell migration and wound closure in vitro. FLS migration rates were calculated by measuring cell coverage into the defect space at defined time points following initial cruciform injury (Figure 5A–H). Compared to untreated controls, a gradual improvement in cell migration was observed in FLS treated with PEMF stimulation with a significant increase in wound closure percentage at 24 h (*p* = 0.0319) and 48 h (*p* = 0.0007) following injury (Figure 5I). At the 24 h timepoint, FLS exposed to PEMF exhibited increased wound closure with a percent difference of 23% compared to controls. The percent difference in wound closure remained consistent at 48 h following injury with a 20% increase in PEMF treated groups.

### Study 2. Evaluate the effect of DC EF stimulation on FLS migration within a collagen hydrogel matrix via galvanotaxis.

**Figure 6.**
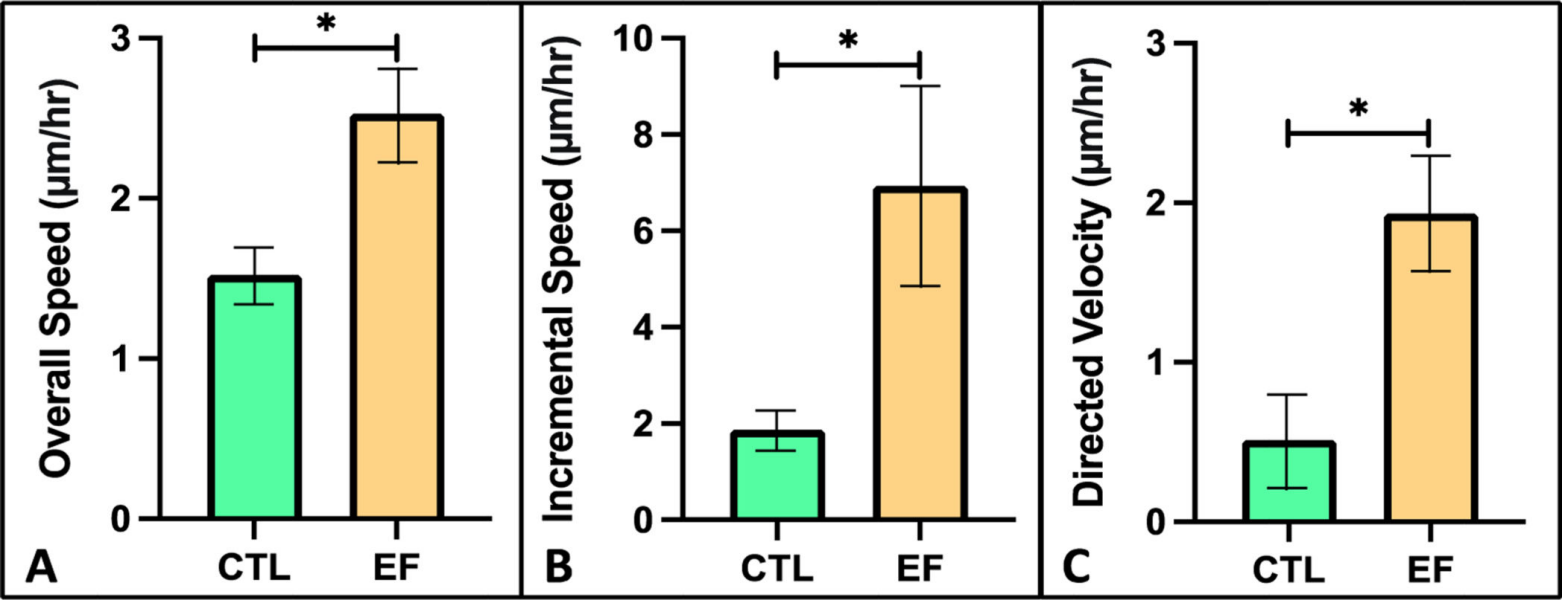
Effect of DC EFs on (**A**) overall speed, (**B**) incremental speed, and (**C**) directed velocity of bovine FLS within a 3D collagen hydrogel (* *p* < 0.001). Parameters were determined by following the path of individual cells for both CTL (*n* = 20 cells) and EF treated (*n* = 24 cells) groups.

**Figure 7.**
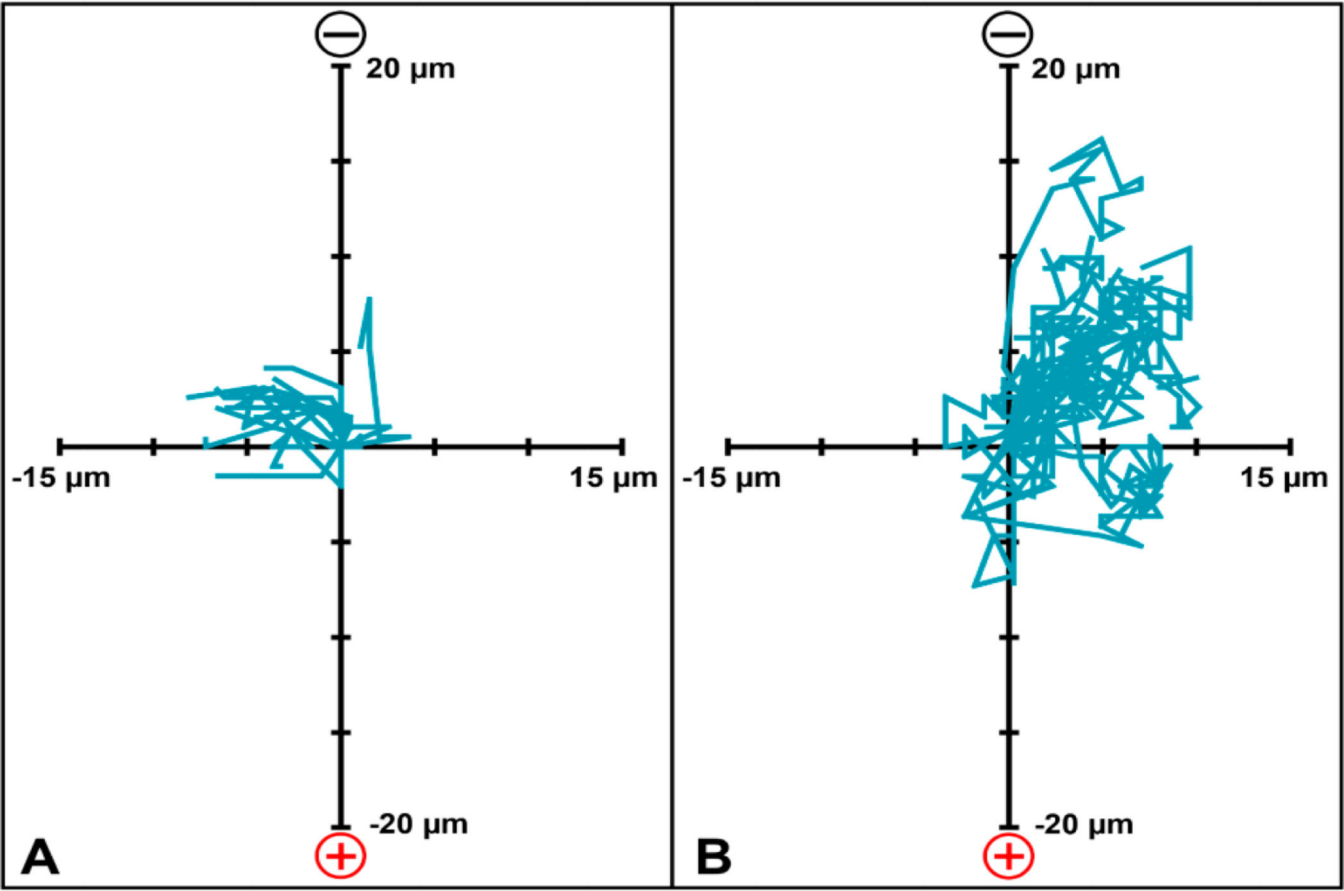
Individual migration paths for (**A**) CTL (*n* = 20) and (**B**) EF (*n* = 24) treated FLS within the 3D collagen gel. Tracking of each cell began at the origin, with positive y-displacement indicating cathodal migration.

Following 3 h of DC EF application, photomicrograph analysis of the FLS-seeded collagen hydrogels revealed a significant increase of 79% in overall migration speed (2.5 μm/h) compared to untreated controls (1.4 μm/h) (Figure 6A; *p* < 0.001). A 3.6-fold increase in average incremental migration speed was observed in FLS under DC EF stimulation (6.9 μm/h) compared to controls (1.9 μm/h) following each 10 min acquisition interval (Figure 6B; *p* < 0.001). Directed velocity of FLS migration within the hydrogels toward the cathode was significantly higher with galvanotaxis EF stimulation versus controls (1.9 and 0.5 μm/h, respectively) (Figure 6C; *p* < 0.001). Monitoring the path of each individual cell revealed that the mean FLS migration angle was 46° for the DC EF exposed hydrogels compared to 156° for the untreated control group (Figure 7).

### Study 3. Apply DC EFs via galvanotaxis to synovium explants in order to evaluate migration of synovial repair cells within a 3D cartilage defect model.

**Figure 8.**
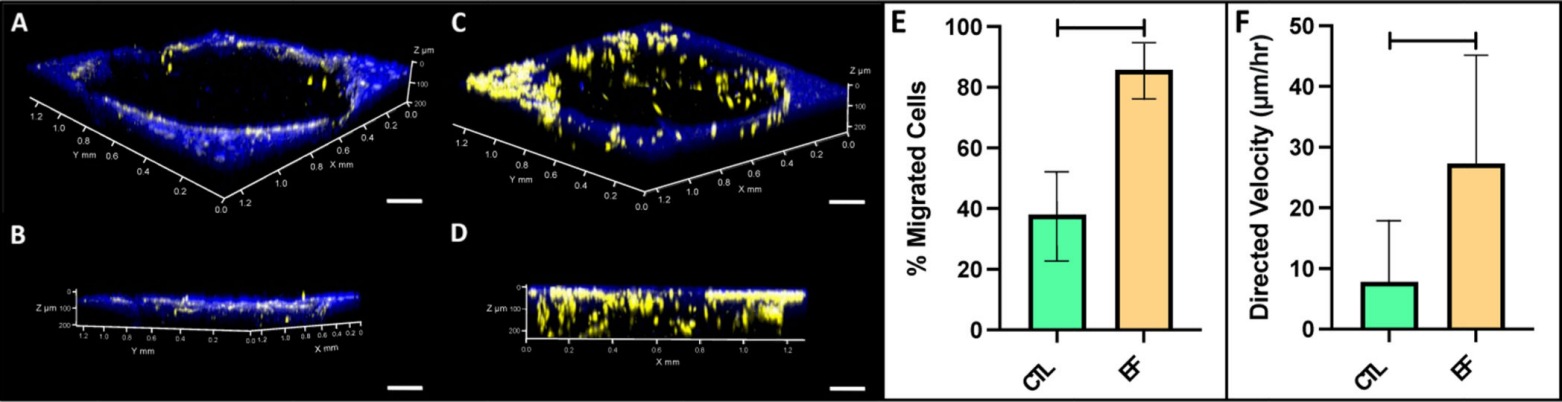
Representative confocal images of DiI-stained FLS migrating into DAPI-labeled bovine cartilage defect. (**A**) Top and (**B**) side view of control (CTL) explants showing minimal FLS migration into the cartilage construct. (**C**) Top and (**D**) side view of explant exposed to DC EFs for 3 h showing significant FLS migration through the depth of the defect region (Scale bar = 200 μm). (**E**) Percent migration and (**F**) directed velocity of FLS into the cartilage defect for CTL and EF treated groups (*n* = 4 cartilage explants).

DC EF stimulation via galvanotaxis increased cell recruitment and migration within the bovine cartilage defect (Figure 8C,D) compared to controls (Figure 8A,B). Assessing the depth of the defect region following DC EF exposure, approximately 86% of adherent cells had migrated at least 40 μm in the EF group, compared to 38% in the controls (Figure 8E; *p* < 0.001). Additionally, EF stimulation increased the directed velocity of FLS into the defect site (28.2 μm/h) relative to untreated controls (8.9 μm/h) (Figure 8F; *p* < 0.001).

**Figure 9.**
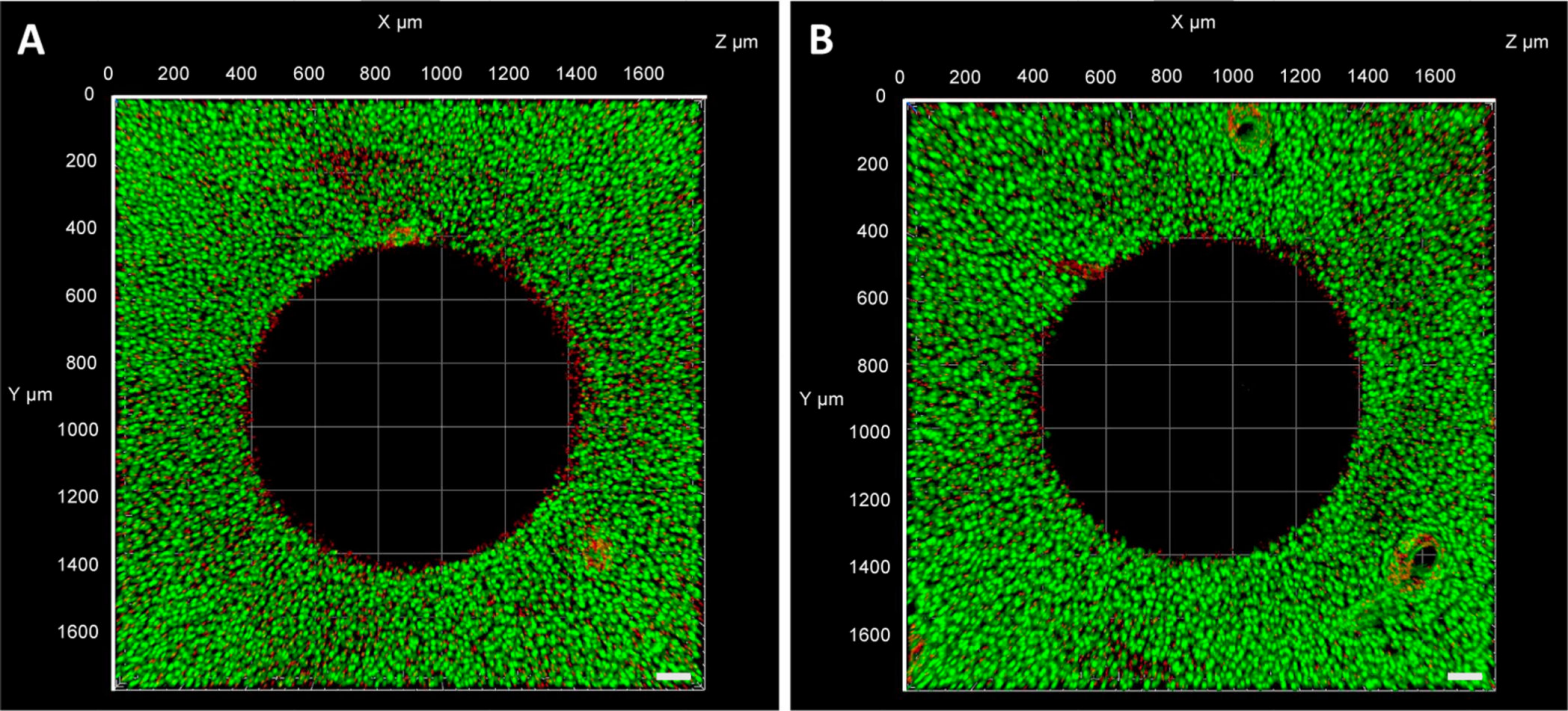
Representative live/dead images of (**A**) control and (**B**) DC EF treated cartilage explants following DC EF stimulation for 3 h (Scale bar = 100 μm).

Following DC EF exposure, viability of the cartilage construct was also assessed using a live/dead cytotoxicity assay. The assay is based on Calcein AM permeating the cells with intact membranes, producing a green fluorescence (live), while Ethidium Homodimer permeates the nuclei of dead cells to produce a red fluorescence (dead) [49]. Compared to untreated controls from initial harvest (Figure 9A), viability of the cartilage construct remained consistent with minimal dead cells under DC EF exposure (Figure 9B). Dead cells were visible along the circumference of the annulus region where the 1 mm sub-core was removed from the cartilage construct. The ring of dead cells was not as profound in the cartilage explants exposed to EF stimulation compared to control samples.

### Study 4. Characterize the effect of PEMF stimulation on modulating the migration of endogenous and/or exogenous FLS repair cells into a cartilage defect model.

**Figure 10.**
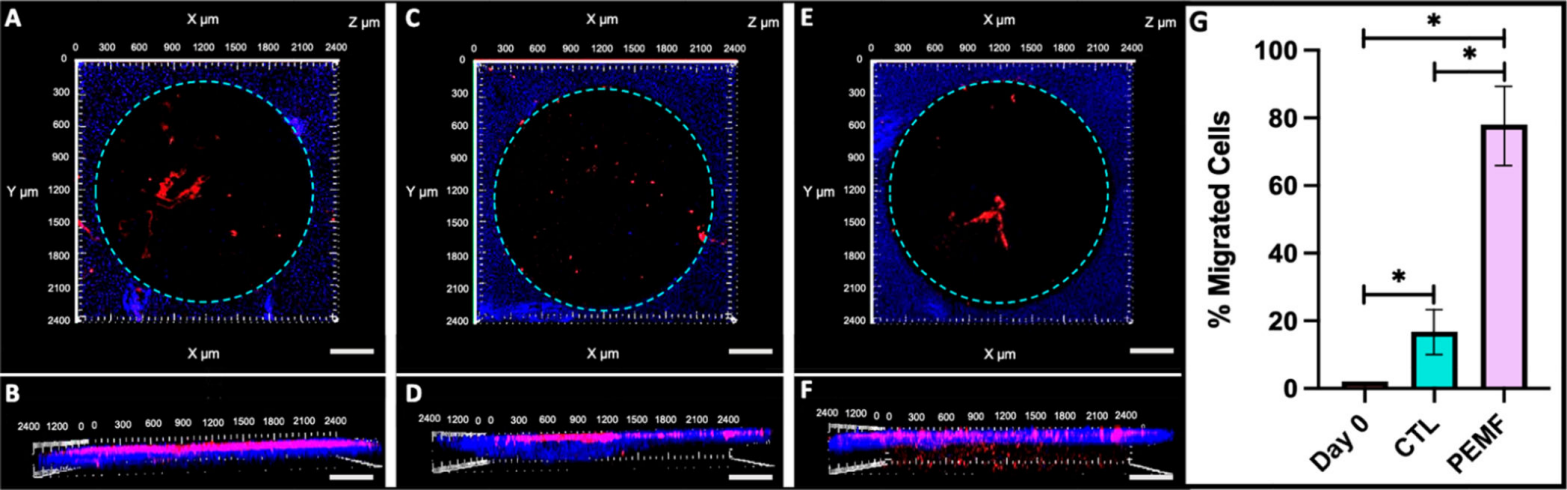
Representative confocal images of DiI-stained FLS migrating into DAPI-labeled cartilage defect. (**A**) Top and (**B**) side view of day 0 samples showing no FLS migration. (**C**) Top and (**D**) side view of control group (CTL) without PEMF stimulation for 48 h showing minimal cell migration (**E**) Top and (**F**) side view of explant exposed to PEMF for 48 h showing significant migration through the cartilage depth (Scale bar = 200 μm). (**G**) Quantified cell count of FLS migration into the cartilage defect across day 0, CTL, and PEMF treated explants (* *p* < 0.001).

PEMF stimulation for 48 h enhanced the migration of FLS into the cartilage defect region compared to untreated controls across both timepoints (Figure 10A–F). The number of migrated FLS was significantly elevated under PEMF exposure, where 78% of adherent cells had migrated at least 40 μm into the defect area compared to 17% in the sham group (Figure 10G; *p* < 0.001).

**Figure 11.**
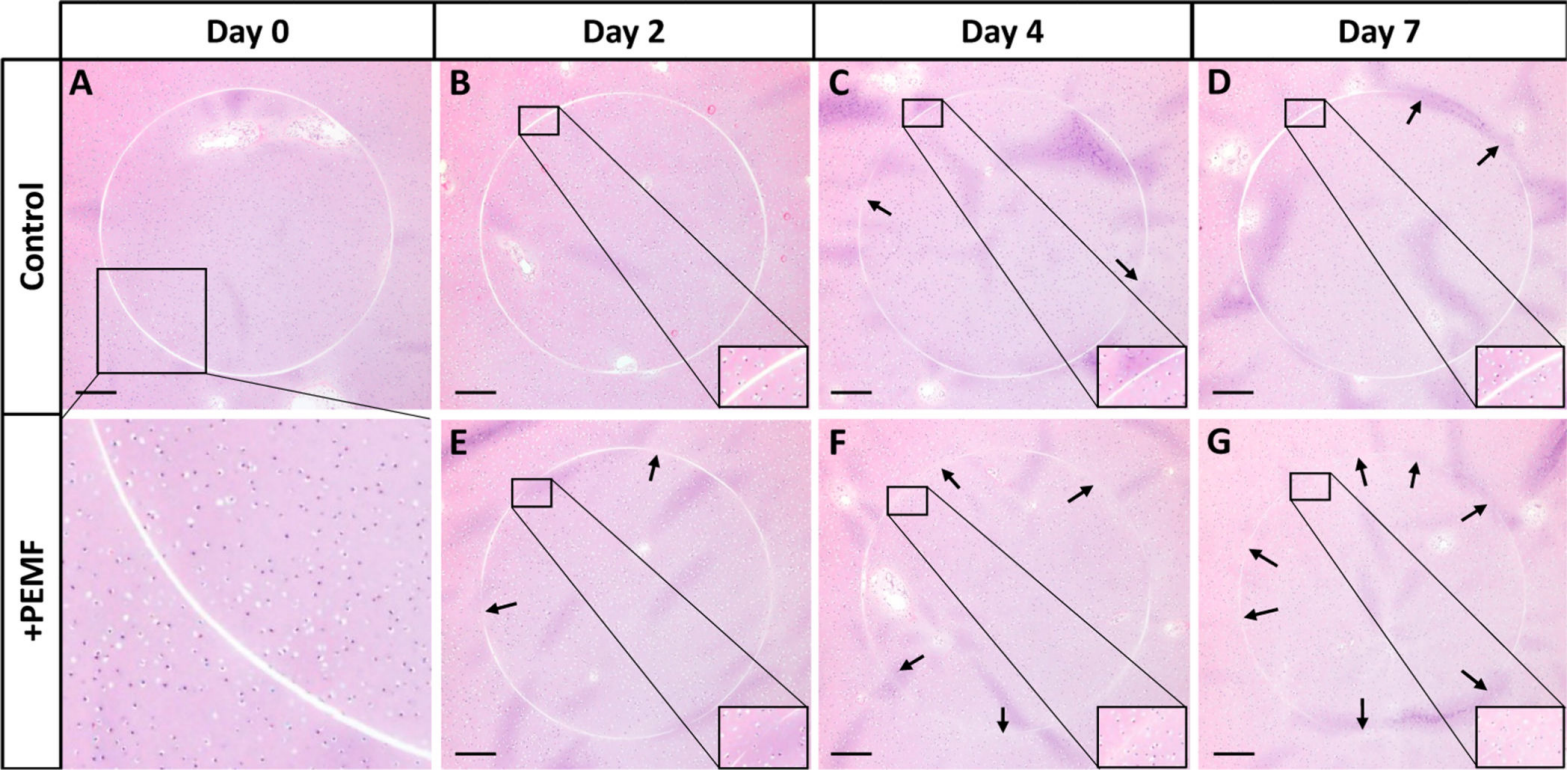
H&E-stained bovine cartilage explants to assess defect repair for (**A–D**) control and (**E–G**) PEMF treated samples (Scale bar = 300 μm). A uniform defect region was formed by removing 1 mm subcores and reinserting the annuli into the cartilage explant. Arrows indicate areas of closure along the cell-free defect region.

Histological staining of the synovium on cartilage explant model was performed to further assess cell migration into the defect site. In this case, the 1 mm cores that were removed from each 4 mm bovine cartilage plug were reinserted in order to characterize intrinsic repair of the defect region via PEMF-induced cell migration. A piece of bovine synovium was placed in direct apposition to the cartilage construct and the explants were exposed to PEMF treatment for up to 7 days. Structural morphology of the cartilage plugs and the migration of FLS as well as native chondrocytes were visualized with H&E staining. Compared to controls (Figure 11A–D), PEMF treatment slightly improved cell migration within the defect region of the cartilage construct over time (Figure 11E–G). The defect ring at the sub-cored interface visually showed enhanced cellular migration and repair by day 7 of PEMF stimulation compared to day 0 controls, as the cell-free defect region exhibited gradual closure across each timepoint.

### Study 5. Evaluate the biochemical properties and ECM components of synovium and cartilage explants exposed to PEMF treatment.

**Figure 12.**
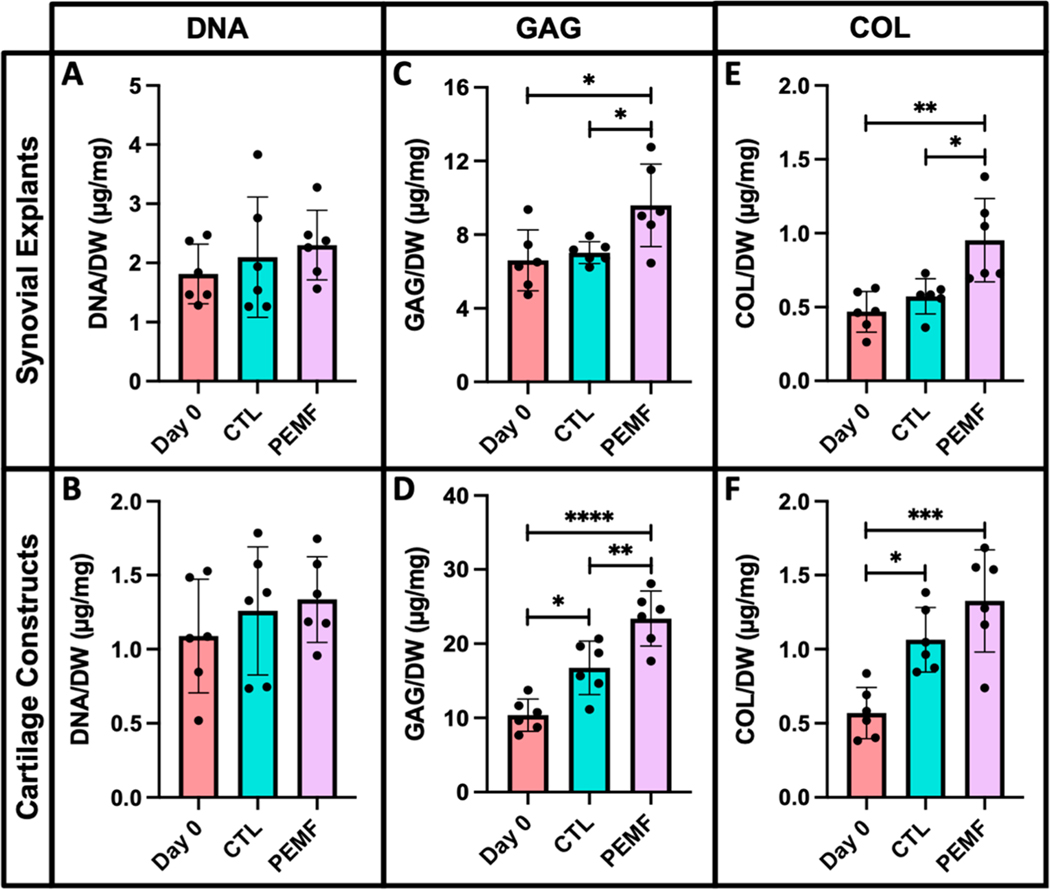
Normalized (**A,B**) DNA, (**C,D**) GAG, and (**E,F**) collagen to respective sample dry weight for PEMF treated synovium and cartilage explants compared to controls (* *p* < 0.05, ** *p* < 0.01, *** *p* < 0.001, **** *p* < 0.0001).

In bovine synovium and cartilage explants, no significant changes in DNA levels were observed between PEMF treated groups and controls (Figure 12A,B). Synovium exposed to PEMF exhibited a 37% increase in GAG content compared to untreated day 7 samples (Figure 12C; *p* = 0.0406). In cartilage constructs, the difference in GAG was slightly more profound for PEMF treated explants with an overall increase of 40% relative to day 7 controls (Figure 12D; *p* = 0.0076). Compared to explants collected during initial harvest (day 0), PEMF exposed synovium exhibited a 45% increase in GAG levels (*p* = 0.0173). Both day 7 controls and PEMF treated cartilage explants also had significantly elevated GAG compared to day 0 samples (*p* = 0.0101 and *p* < 0.0001, respectively). Synovium under PEMF treatment also exhibited a 66% increase in collagen content compared to day 7 controls (*p* = 0.0103), while no significant changes were observed between the same treatment groups for the cartilage explants (Figure 12E,F). Compared to day 0 specimens, a greater than 2-fold increase in collagen levels was observed for both synovium and cartilage tissue under PEMF stimulation (*p* = 0.0016 and *p* = 0.0003, respectively). In cartilage explants, an 87% increase in collagen levels were also measured in day 7 controls compared to day 0 samples (*p* = 0.0117).

**Figure 13.**
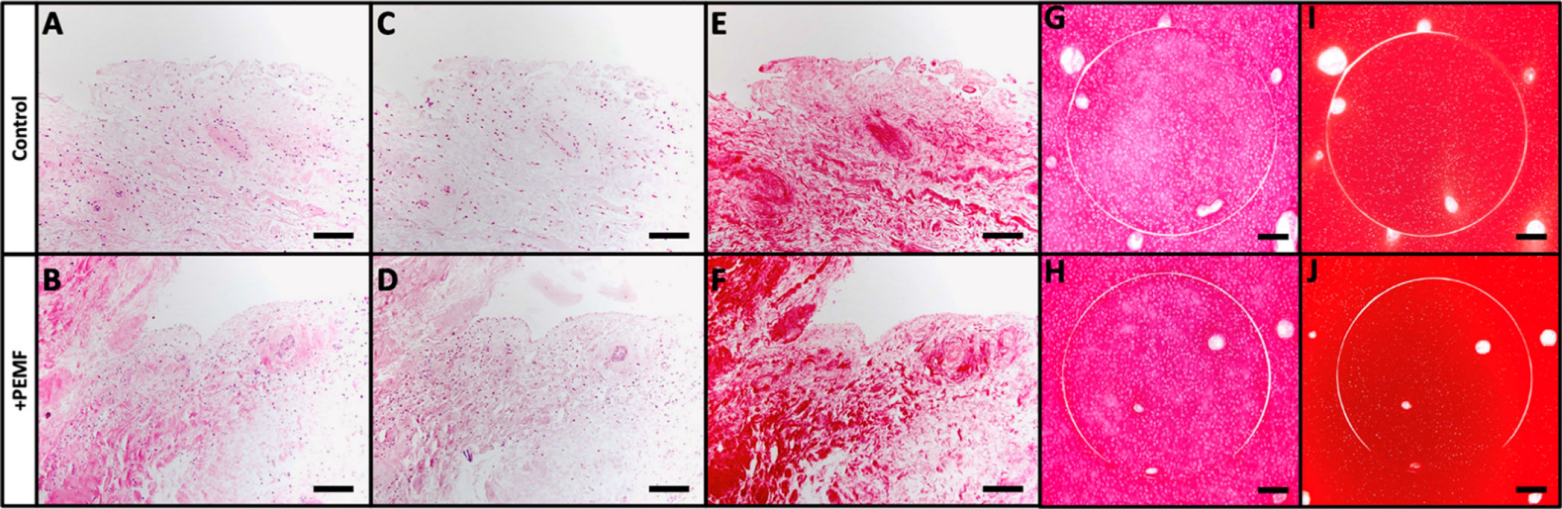
Histological staining of PEMF treated explants. (**A,B**) H&E, (**C,D**) Safranin O, and (**E,F**) Picrosirius Red staining of bovine synovium explants (Scale bar = 100 μm). (**G,H**) Safranin O and (**I,J**) Picrosirius Red staining of 4 mm bovine cartilage plugs with 1 mm subcores that were reinserted into the explant forming a circular defect region (Scale bar = 400 μm).

Histological staining of PEMF treated synovium and cartilage explants yielded similar results. Similarities in synovial structural morphology between PEMF and control samples were visualized with H&E (Figure 13A,B). Slightly deeper staining of Safranin O was observed in both PEMF treated synovial and cartilage explants compared to untreated controls (Figure 13C,D,G,H). Higher intensity Picrosirius Red staining was also evident in synovium exposed to PEMF stimulation (Figure 13E,F), while no visual differences were observed between the cartilage constructs (Figure 13I,J).

**Figure 14.**
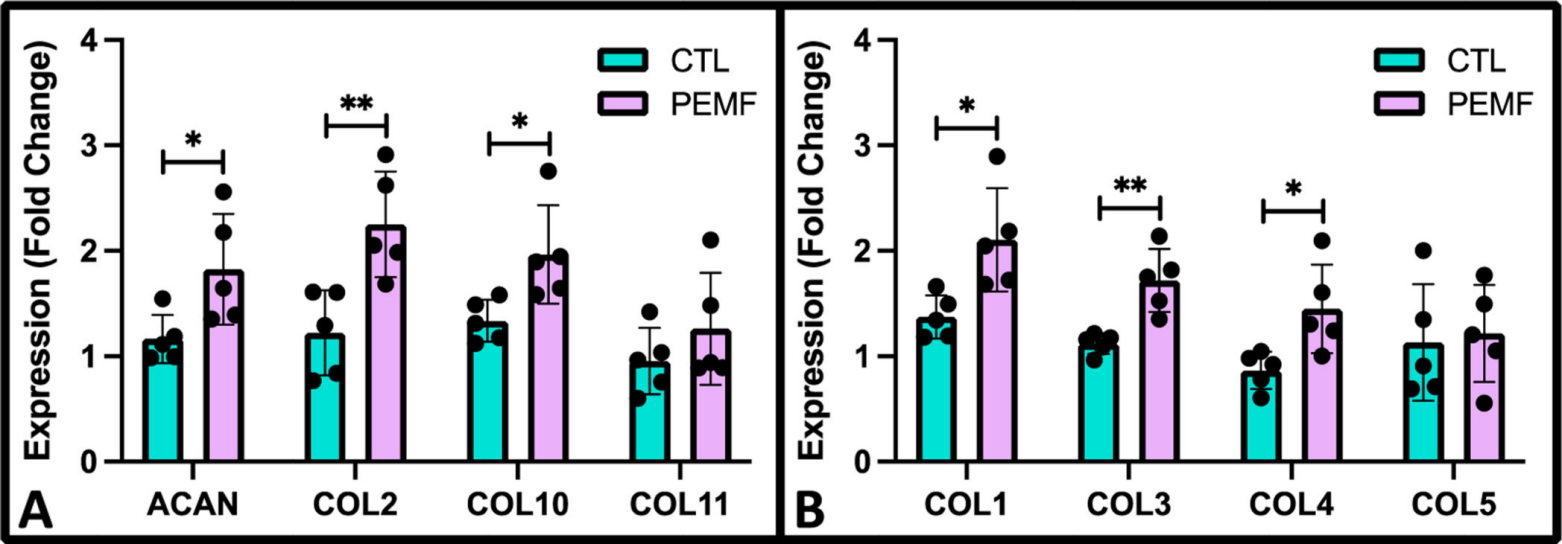
Gene expression of bovine cartilage explants normalized to day 0 controls using (**A**) cartilage (ACAN, COL2, COL10, COL11) and (**B**) synovium (COL1, COL3, COL4, COL5) ECM markers following 7 days of PEMF stimulation with parallel untreated controls (*n* = 6; * *p* < 0.05, ** *p* < 0.01).

Gene expression of PEMF exposed bovine cartilage explants showed significant differences in both cartilage and synovium ECM markers compared to untreated controls. Following 7 days of PEMF stimulation, cartilage makers of ACAN, COL2, and COL10 were significantly upregulated (Figure 14A; *p* = 0.0329, *p* = 0.0073, and *p* = 0.0243, respectively). Compared to controls, synovium ECM markers including COL1, COL3, and COL4 were also elevated under PEMF exposure (Figure 14B; *p* = 0.0151, *p* = 0.0029, and *p* = 0.0210, respectively).

## 4. Discussion

The presented studies characterized the use of PEMFs and DC EF galvanotaxis to promote the migratory behavior and healing capacity of native bovine FLS. The bovine model is widely used in musculoskeletal research and has yielded significant insights to cartilage biology, synovial activation, and regenerative medicine. Previous studies from our laboratory have demonstrated that bovine synovium (engineered and native) respond similarly to pro-inflammatory cytokines as native human synovium, supporting future efforts to develop more effective strategies for promoting repair and restoring joint health [46].

Electrotherapeutic stimulation is yet to be fully explored in the context of promoting intrinsic cartilage repair via FLS modulation. Given its proximity to the underlying cartilage, synovium-derived cells have been implicated in localized repair of injured lesions along the articular surface [50–52]. Combined with the use of electrotherapeutic strategies, direct homing of resident FLS can further be enhanced, thus expediting the rate of cartilage repair without surgical interventions.

Compared to untreated controls, PEMF exposure enhanced bovine FLS migration during wound closure, suggesting that EF stimulation can promote FLS movement in vitro. While no immediate effects of PEMF were observed directly following injury, sustained improvement in the rate of FLS wound closure was evident by 24 h, contributing to current literature findings that support the use of PEMFs in modulating gradual migration of other cell types including MSCs, chondrocytes, osteoblasts, and meniscal derived cells [37,53–57].

From a 2D environment, the migration of bovine FLS was further investigated within a 3D collagen hydrogel via galvanotaxis. In the collagen substrate, bovine FLS exhibited migration toward the cathode with increased incremental and overall speeds of movement, suggesting that DC EF stimulation can promote the directed migration of FLS under 3D culture conditions. In this study, FLS migration was tracked by identifying the centroid and tracing the path followed by each cell within the collagen hydrogel. However, this method of cell tracking may have underestimated the displacement of the cells in the galvanotaxis chamber. FLS may have formed additional focal matrix adhesions with the surrounding collagen gel, potentially contributing to delayed migration rates [58,59].

Following FLS migration within a 3D collagen hydrogel, a custom galvanotaxis chamber was designed in order to assess cell movement within a biological substrate. EF strengths of magnitudes similar to those used to promote 2D galvanotaxis was applied to track cell migration into the defect of the bovine cartilage explant. FLS migrated into the defect region at an average velocity of 28.2 μm/h with an EF strength of 6 V/cm and a current density equal to 73 mA/cm^2^. These values are comparable to physiologic conditions exhibited by chondrocytes in vivo, where cells can be exposed to field strengths and current densities with magnitudes of up to 15 V/cm and 100 mA/cm^2^, respectively [60]. The EF parameters of this study were optimized to preserve FLS viability under the tested conditions while also maximizing DC EF exposure to promote migration, suggesting that novel bioreactors can be designed to investigate 3D tissue specimens and subsequent cell migration analysis under galvanotaxis stimulation.

Following a similar procedure, PEMF contributed to increased FLS migration into the bovine cartilage defect region, suggesting that PEMF exposure can promote intrinsic repair. This finding supports previous studies that have shown PEMF treatment improves cartilage graft growth and healing in both a time- and direction-dependent manner [37]. H&E staining of the defect region reveled improved repair under PEMF following 7 days of EF exposure. Whether the repair process was initiated by FLS from the overlying synovium or native chondrocytes from the articular cartilage surface is yet to be determined. However, recent studies have shown that PEMF treatment can induce MSC differentiation to promote immunomodulation and improve cartilage regeneration in vitro and in vivo [53]. The multi-lineage differentiation capabilities of MSCs combined with PEMF stimulation have been shown to promote osteogenesis and chondrogenesis in joint repair [54,57]. With appropriate stimulation, FLS have also been shown to possess a multi-lineage potential to differentiate into chondrocytes and exhibit characteristics of the native cartilaginous environment [61,62]. The accelerated repair of the bovine cartilage defect region in the current study may be induced by a combination of PEMF stimulated chondrocyte migration and potential chondrogenesis of native FLS into the injured site, contributing to an improved healing response compared to untreated controls. PCR analysis of PEMF treated explants revealed that gene expression of chondrogenic and synovium ECM markers were upregulated compared to no-PEMF controls. These results may reflect the contribution of gene expression from cells recruited to the wound site and those derived from the entirety of the cartilage construct.

PEMF-induced chondrogenesis in situ may further contribute to increased cartilage ECM constituents in injured joints [53]. Biochemistry analysis revealed that DNA levels were consistent between PEMF and control treated specimens, suggesting that EF stimulation did not induce cell damage for both synovium and cartilage explants. Collagen and GAG content differed significantly between PEMF and untreated samples, suggesting that EF stimulated environments may affect cell sensitivity and metabolism [43,63]. While lower GAG and collagen content have been shown to be characteristic features associated with cartilage damage, PEMF treatment contributed to elevated matrix constituents for both bovine synovium and cartilage tissue, indicative of its pro-anabolic effect [34,64–68]. Histological analysis of both explants confirmed the differences observed in GAG and collagen levels under PEMF exposure compared to controls.

Future studies will aim to optimize PEMF and DC EF culture conditions. While the current galvanotaxis set-up did not allow for examination of bi-directional cell migration, the system can be modified by culturing labeled FLS on multiple surfaces of the cartilage hydrogel or explant model to further investigate preferential migration towards the cathode (−). In addition, mechanisms that mediate differences in cell attachment versus interactions between the examined substrates can be distinguished in order to confirm the role of PEMFs and DC EF stimulation in promoting FLS migration. While the present PEMF and galvanotaxis systems were unable to perform real-time cell tracking, both procedures allowed for the maintenance of aseptic conditions, multiple treatments over time, and subsequent cell and tissue analyses.

Overall, both PEMFs and galvanotaxis DC EF treatments can enhance synovial cell-mediated cartilage repair using clinically relevant stimulation parameters. The results suggested that PEMF and galvanotaxis are complementary electrotherapeutic strategies to promote the migration of FLS repair cells within cartilage ECM or toward defect regions along the articular surface, thus initiating an intrinsic healing response. The novel tissue-scale bioreactor was designed to generate consistent DC EFs under sterile culture conditions to investigate the effects of galvanotaxis on synovium and cartilage explants. Similarly, custom PEMF generators were designed to test 3D biological specimens under constant EF exposure in order to examine the metabolic and migratory behavior of FLS within an in vitro cartilage defect model. Together, PEMF and galvanotaxis DC EF stimulation provide electrotherapeutic modalities to translate culture findings to a preclinical system.

## Figures and Tables

**Table 1.** qPCR Primer Sequences for cartilage and synovium markers. ACAN: Aggrecan, COL1: collagen type I, COL2: collagen type II, COL3: collagen type III, COL4: collagen type IV, COL5: collagen type V, COL10: collagen type X, COL11: collagen type XI, GAPDH (housekeeping gene).

Gene	FWD Primer Sequence	REV Primer Sequence
GAPDH	GTC ATC ATC TCT GCA CCT TCT G	GGA GGC ATT GCT GAC AAT CT
COL10A1	CTG CCC GAG GAC TTT GTA A	GGG TAA GCT TTG GAG AGG ATA A
COL2A1	GGC CTG TCT GCT TCT TGT AA	CTA GAG TGA CTG GGA TTG GAA AG
COL1A2	TCC AGA AGG CTC TAG GAA GAA	CTT GGT TAG GGT CAA TCC AGT AG
COL11A2	CCT GGA TGA GGA AGT CTT TGA G	CTT CTG GTC ACA GGA TTC GTA G
ACAN	CCT CAG GGT TTC CTG ACA TTA G	GCT CAG TCA CGC CAG ATA TT
COL3A1	TGG TAT TCC TGG GCG AAA TG	CCA CCA GTA GGA CAT GAT TCA C
COL1A1	CAG ACT GGC AAC CTC AAG AA	TAG GTG ACG CTG TAG GTG AA
COL4A1	CAC GGC TAC TCT TTG CTC TAC	GAA GGG CAT GGT ACT GAA CTT

## Data Availability

The datasets analyzed in this study are available from the corresponding authors on reasonable request.
